# Beyond hot flashes: Exploring the role of estrogen therapy in postmenopausal women for myocardial infarction prevention and recovery

**DOI:** 10.17305/bb.2023.9535

**Published:** 2024-02-01

**Authors:** Aakash Choradia, Karoona Bai, Suha Soni, Nhan Nguyen, Shikha Adhikari, Dalween Kaur Rahul, Rahul Gupta

**Affiliations:** 1 Kist Medical College, Kathmandu, Nepal; 2 Dow Medical College, Karachi, Pakistan; 3 University of Texas Health Science Center School of Public Health, Texas, USA; 4 University of Debrecen, Debrecen, Hungary; 5 University College of Medical Sciences, New Delhi, India; 6 Malaysian Medical Association, Malaysia; 7 Independent Researcher, Khartoum, Sudan

**Keywords:** Estrogen therapy, postmenopausal women, myocardial infarction (MI) prevention, cardiovascular health, hormone replacement therapy (HRT)

## Abstract

Myocardial infarction (MI) commonly known as “heart attack” results from the blockage of blood flow to the heart. Postmenopausal women face an elevated risk of MI due to declining estrogen levels, a hormone pivotal in maintaining cardiovascular health. It promotes vasodilation, reduces inflammation, and improves lipid profiles. While estrogen therapy shows promise in mitigating MI risk for postmenopausal women, its efficacy in prevention and recovery remains a subject of debate. This review provides a critical assessment of existing evidence on estrogen therapy’s cardioprotective effects for postmenopausal women. It delves into estrogen’s role in vascular function enhancement, inflammation reduction, and lipid metabolism modulation. Additionally, it addresses the various forms of estrogen therapy, administration methods, dosage considerations, safety implications, and associated risks. The review highlights the existing controversies and knowledge gaps related to estrogen therapy for MI prevention. It underscores the urgency for in-depth research to decipher the nexus between estrogen therapy and MI risk, especially concerning primary prevention and specific postmenopausal subgroups. Future studies should investigate optimal formulations, doses, and administration routes of estrogen therapy as well as assess treatment timing and duration. Comparative studies and long-term follow-up are necessary to inform clinical decision making and improve patient care. Addressing these research gaps will empower clinicians to make more judicious choices about estrogen therapy for MI prevention and recovery in postmenopausal women, aiming for enhanced patient outcomes.

## Introduction

Myocardial infarction (MI), commonly known as a “heart attack,” occurs when blood flow to the myocardium is partially or completely blocked. This condition can range from being silent and unnoticed to a catastrophic event leading to a decline in heart function and even sudden death [1]. The primary cause of most MIs is coronary artery disease, which results in prolonged oxygen deprivation, leading to the death and necrosis of myocardial cells [2]. Symptoms may include chest discomfort or pressure, which can radiate to the neck, jaw, shoulder, or arm. The diagnosis involves evaluating the patient’s medical history, conducting a physical examination, and using diagnostic tools like alterations in electrocardiogram (ECG) and elevated cardiac troponin levels [3, 4].

Postmenopausal women face a higher risk of developing MI than their premenopausal counterparts [5, 6]. Menopause, marked by a decline in ovarian function, results in a significant reduction of endogenous estrogen levels. This reduction can adversely affect cardiovascular health. The increased incidence of MI in postmenopausal women underscores the influence of menopause on cardiovascular risk [5, 6]. The loss of estrogen’s protective effects on the cardiovascular system is believed to contribute to this heightened risk. Estrogen plays a crucial role in maintaining vascular health by promoting vasodilation, reducing inflammation, and improving lipid profile [7, 8]. Consequently, the decline in estrogen levels during menopause can lead to unfavorable changes in these cardiovascular parameters, potentially contributing to the increased risk of MI in postmenopausal women [9].

The gold standard for preventing and managing MI involves a comprehensive approach that encompasses lifestyle changes, pharmacological interventions, and cardiac rehabilitation programs. Lifestyle modifications include adopting heart-healthy dietary habits, engaging in regular physical activity, quitting smoking, and managing weight [10]. Tailored pharmacological interventions based on individual risk factors comprise the use of antiplatelet agents, beta-blockers, statins, and ACE inhibitors/ARBs [11]. Cardiac rehabilitation programs offer structured physical activity, education, psychological support, and risk factor management [12].

Given the impact of declining estrogen levels during menopause on cardiovascular health, estrogen therapy has emerged as a potential intervention to reduce the risk of MI in postmenopausal women [13]. Estrogen has beneficial effects on the cardiovascular system, including vasodilation, anti-inflammatory properties, improvement in lipid profile, and modulation of plaque stability [7, 8]. Understanding the rationale for using estrogen therapy provides insight into the potential benefits and drawbacks of this approach. Thus, this study aims to critically assess the existing evidence regarding the role of estrogen therapy in postmenopausal women for the prevention and recovery from MI, weighing both its cardiovascular benefits and potential risks. Our goal is to enrich the current understanding of estrogen therapy in this setting and guide clinical decision making for patients.

## Estrogen and cardiovascular health

### Mechanisms of estrogen’s cardioprotective effects on vascular function

Estrogen exerts multiple cardioprotective effects on vascular function through complex mechanisms. First, it plays a pivotal role in promoting vasodilation, which expands blood vessels and boosts blood flow [14]. This vasodilatory effect results from an increase in the availability and release of nitric oxide, a powerful vasodilator [14]. Nitric oxide acts on the smooth muscle cells that line the blood vessels, prompting them to relax, which in turn enhances endothelial function. Consequently, estrogen contributes to optimal blood flow and vascular health by facilitating vasodilation.

Numerous studies have demonstrated that estrogen stimulates endothelial nitric oxide synthase (eNOS) expression and activity, which results in increased production of nitric oxide [14]. Furthermore, estrogen promotes eNOS coupling, favoring the production of nitric oxide over superoxide anions, reinforcing its vasodilatory effects [14]. These findings elucidate the molecular basis of estrogen’s influence on vasodilation and its potential value as a therapeutic target for cardiovascular health.

Second, estrogen modulates the renin–angiotensin–aldosterone system, a complex hormonal cascade involved in blood pressure regulation and cardiovascular homeostasis [15]. Estrogen inhibits the production of angiotensin II, a potent vasoconstrictor, thereby affecting this system [15]. By reducing angiotensin II levels, estrogen facilitates vasodilation and diminishes vascular resistance [15]. This vasodilatory action aids in maintaining normal blood pressure and bolsters overall cardiovascular function.

Numerous experimental studies have investigated the role of estrogen in modulating the renin–angiotensin–aldosterone system. Estrogen has been found to reduce the expression and activity of angiotensin-converting enzyme (ACE), a key enzyme responsible for the conversion of angiotensin I to angiotensin II [16]. Additionally, estrogen upregulates the expression of angiotensin-converting enzyme 2 (ACE2), an enzyme that converts angiotensin II to angiotensin-(1-7), a peptide with vasodilatory and anti-inflammatory properties [16].

These changes in the renin–angiotensin–aldosterone system contribute to estrogen’s vasodilatory effects and its potential as a therapeutic approach for cardiovascular diseases.

**Figure 1.**
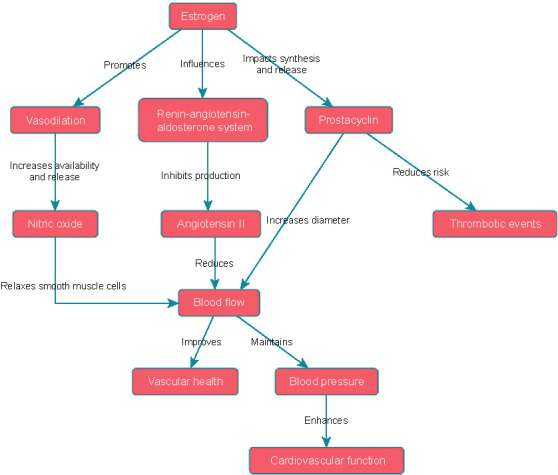
Mechanisms of estrogen’s cardioprotective effects on vascular function.

Furthermore, estrogen influences the synthesis and release of prostacyclin, a crucial molecule with vasodilatory and antithrombotic properties [17]. Prostacyclin acts as a potent vasodilator by relaxing the smooth muscle cells in the walls of blood vessels, leading to an increased blood vessel diameter and improved blood flow [18]. Moreover, it inhibits platelet aggregation and clot formation, thereby reducing the risk of thrombotic events [18]. The promotion by estrogen of prostacyclin synthesis and release contributes to enhanced vascular health and a reduced tendency for blood clot formation (Figure 1).

Studies have shown that estrogen upregulates the expression of cyclooxygenase-2 (COX-2), the enzyme responsible for prostacyclin synthesis, in endothelial cells [ref]. Estrogen receptor-mediated signaling pathways are implicated in this COX-2 upregulation, indicating a direct involvement of estrogen in promoting prostacyclin production [19].

Understanding these mechanisms deepens our insight into estrogen’s role in vascular function and its potential therapeutic implications for cardiovascular diseases.

### Effects of estrogen on inflammation and lipid metabolism

Estrogen exerts significant effects on inflammation and lipid metabolism, both of which are crucial to cardiovascular health. Firstly, estrogen demonstrates potent anti-inflammatory properties by regulating the immune response and suppressing the production of pro-inflammatory cytokines and adhesion molecules [20, 21]. This inhibition helps mitigate chronic inflammation and reduces the risk of endothelial dysfunction, a key contributor to the development of atherosclerosis [20]. Additionally, estrogen inhibits the adhesion of inflammatory cells to the endothelium, preventing their infiltration into arterial walls and subsequent plaque formation [22].

The anti-inflammatory effects of estrogen are mediated through various mechanisms. Estrogen can inhibit the activation of nuclear factor-kappa B (NF-κB), a critical transcription factor involved in the expression of pro-inflammatory genes, via both estrogen receptor-dependent and -independent pathways [23]. Estrogen receptor signaling has been demonstrated to directly interact with NF-κB, inhibiting its nuclear translocation and consequently reducing the transcription of pro-inflammatory genes [23]. Furthermore, estrogen can enhance the production of anti-inflammatory cytokines, such as interleukin-10 (IL-10), further attenuating the inflammatory response [21]. These findings highlight the intricate and multifaceted nature of estrogen’s anti-inflammatory actions and its potential as a therapeutic agent for inflammation-related cardiovascular diseases.

**Figure 2.**
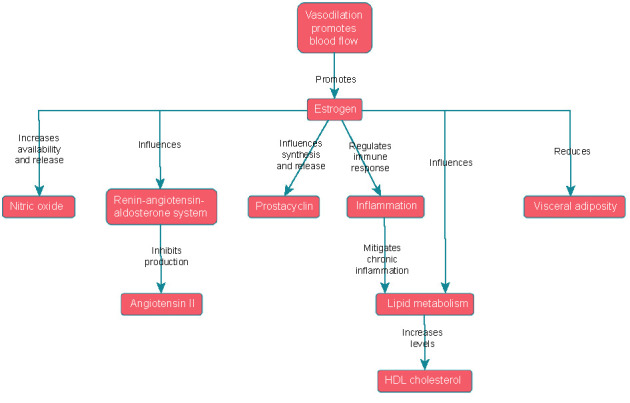
Effects of estrogen on inflammation and lipid metabolism.

Secondly, estrogen influences lipid metabolism in a way that favors cardiovascular health. It promotes favorable lipid profiles by increasing levels of high-density lipoprotein (HDL) cholesterol, commonly known as “good” cholesterol [24]. HDL cholesterol plays a pivotal role in reverse cholesterol transport, which entails the removal of low-density lipoprotein (LDL) cholesterol, or “bad” cholesterol, from peripheral tissues, including arterial walls [24]. By aiding the transport of excess cholesterol from the arteries to the liver for metabolism and elimination, HDL cholesterol helps prevent the build-up of cholesterol-rich plaques, thereby reducing the risk of atherosclerosis. Estrogen’s capability to elevate HDL cholesterol levels leads to enhanced lipid metabolism and diminished cardiovascular risk (Figure 2).

The impact of estrogen on lipid metabolism encompasses the regulation of various lipid-related genes and pathways. Estrogen has been demonstrated to increase the expression of the ATP-binding cassette transporter A1 (ABCA1), a pivotal protein involved in HDL cholesterol synthesis and reverse cholesterol transport [25, 26]. Estrogen-mediated activation of liver X receptors (LXRs), nuclear receptors that regulate lipid metabolism, has been linked to the upregulation of ABCA1 expression [27, 28]. Furthermore, estrogen can suppress the expression of pro-inflammatory genes, such as lipoprotein lipase (LPL) and scavenger receptor class B type 1 (SR-B1), both of which are linked to LDL cholesterol accumulation and the onset of atherosclerosis [29]. These insights elucidate the molecular mechanisms by which estrogen modulates lipid metabolism and their potential implications for cardiovascular health.

Furthermore, estrogen influences the distribution of body fat, tending to reduce visceral adiposity, a type of fat linked with heightened inflammation and cardiovascular risk [30, 31]. The effects of estrogen on adipocyte biology have been extensively studied, with research suggesting that estrogen receptor signaling can regulate both adipocyte differentiation and lipogenesis [10]. Estrogen receptors are present in adipocytes, and their activation impacts adipocyte function and lipid storage [10]. These effects underscore estrogen’s capacity to reduce visceral adiposity and its potential significance for cardiovascular health.

### Estrogen’s antiapoptotic and pro-survival effects on cardiomyocytes

Estrogen’s influence on cardiomyocytes goes beyond its beneficial effects on vascular function and lipid metabolism. One pivotal aspect of estrogen’s cardioprotective potential is its anti-apoptotic and pro-survival effects on cardiomyocytes, the specialized heart muscle cells responsible for maintaining cardiac function. Estrogen’s activation of the Akt signaling pathway emerges as a fundamental mechanism that promotes cell survival and inhibits apoptosis in cardiomyocytes [32, 33]. Akt, also known as protein kinase B (PKB), is a serine/threonine kinase integral to regulating cell survival, growth, and metabolism [34].

Estrogen activates Akt through both estrogen receptor-dependent and -independent mechanisms, leading to increased phosphorylation and activation of this kinase. Once activated, Akt phosphorylates and inactivates pro-apoptotic factors, simultaneously promoting the expression of anti-apoptotic proteins. This complex cascade of events, orchestrated by estrogen, bolsters the cardiomyocytes’ resistance to apoptotic cell death, enhancing their survival and function when faced with various stressors [35].

Moreover, estrogen’s inhibitory effect on caspase-3, a central executioner caspase in the apoptotic pathway, further underscores its anti-apoptotic actions [36].

Caspase-3 activation plays a pivotal role in cell death processes, and estrogen has been shown to downregulate its expression and activity in response to apoptotic stimuli. By inhibiting caspase-3, estrogen effectively stops the execution of apoptosis, preserving both the structural and functional integrity of cardiomyocytes during ischemic events, such as MI. This protective mechanism highlights the significance of estrogen’s presence in maintaining cardiac cell viability and forestalling cell death in critical cardiovascular situations [36, 37]. Beyond modulating Akt and caspase-3, estrogen’s anti-apoptotic effects also involve the inhibition of JNK1/2-mediated NF-κB activation [38].

NF-κB is a transcription factor recognized for its multifaceted role in regulating inflammation and cell survival. By inhibiting the activation of JNK1/2, estrogen effectively curtails NF-κB’s pro-inflammatory and pro-apoptotic functions. This fine-tuned control of NF-κB activity by estrogen contributes to the mitigation of inflammation and the protection of cardiomyocytes against apoptotic cell death. This aspect further emphasizes the comprehensive nature of estrogen’s cardioprotective repertoire, encompassing not only vascular benefits but also intricate intracellular pathways that preserve cardiac cell viability [38].

Additionally, estrogen’s pro-survival effects on cardiomyocytes involve its inhibition of glycogen synthase kinase-3 beta (GSK-3β), a serine/threonine kinase critically involved in cell survival and apoptosis regulation [39, 40].

By inhibiting GSK-3β, estrogen promotes cardiomyocyte survival and offers protection against apoptosis triggered by various stress stimuli. This GSK-3β-mediated mechanism adds an additional layer of cardioprotection, enabling cardiomyocytes to withstand multiple challenges and sustain their functionality in the dynamic cardiovascular environment [39].

Furthermore, estrogen’s impact on p38α-mediated p53 phosphorylation represents another crucial aspect of its cardioprotective effects [41].

p53 is a well-known tumor suppressor protein that regulates cell cycle arrest and apoptosis. Estrogen’s inhibition of p38α-mediated phosphorylation of p53 effectively diminishes its transcriptional activity and its pro-apoptotic effects. This orchestrated inhibition of p53 phosphorylation by estrogen offers additional protection to cardiomyocytes, bolstering their resilience under ischemic conditions and reducing the risk of apoptotic cell death [41].

In conclusion, the multifaceted effects of estrogen extend to the realm of cardiomyocytes, where it exerts antiapoptotic and pro-survival influences through intricate signaling pathways. Activation of Akt, inhibition of caspase-3, modulation of JNK1/2-mediated NF-κB activation, inhibition of GSK-3β, and suppression of p38α-mediated p53 phosphorylation are all interconnected mechanisms through which estrogen shields cardiomyocytes from apoptotic cell death. Understanding these intricate cardioprotective mechanisms is vital for unraveling estrogen’s full potential as a therapeutic intervention in MI prevention and recovery among postmenopausal women, leading to enhanced cardiovascular health and overall well-being.

## Estrogen therapy in postmenopausal women

### Types of estrogen therapy available

Estrogen therapy provides postmenopausal women with a variety of options for individualized treatment, tailored to their preferences and unique needs. The most commonly used forms of estrogen therapy include oral formulations, transdermal patches, and topical creams or gels. Each form possesses distinct characteristics, benefits, and considerations that guide the decision making of both healthcare providers and patients (Table 1).

**Table 1:** Comparison of estrogen therapy: type, characteristics, benefits, and considerations

**Type of estrogen therapy**	**Characteristics**	**Benefits**	**Considerations**	**References**
Oral estrogen therapy	– Taken in tablet or pill form – Commonly prescribed: conjugated equine estrogens and synthetic estrogens	– Easy and regular dosing – Suitable for women without skin sensitivities – Convenient dosing regimen	– First-pass metabolism in the liver – Potential increase in clotting factors – Consideration of medical history and risk factors	[21]
Transdermal estrogen patches	– Deliver estrogen directly into the bloodstream – Bypass first-pass metabolism in the liver – Alternative for those with gastrointestinal side effects or concerns about liver metabolism	– Continuous estrogen release – Convenient application (once or twice weekly) – Consistent and steady estrogen release	– Unsuitable for individuals with certain skin conditions or sensitivities	[24]
Topical estrogen creams or gels	– Applied to the skin for systemic delivery – Primarily beneficial for vaginal symptoms such as dryness and discomfort	– Flexible dosing based on individual needs – Non-oral route of administration – Targeted relief from genitourinary symptoms	– Precautions to avoid estrogen transfer through skin contact	[25]

### Oral estrogen therapy

Oral estrogen therapy involves administering estrogen in tablet or pill form. The most commonly prescribed oral formulations include conjugated equine estrogens, derived from pregnant mares’ urine, and synthetic estrogens [42]. This method offers easy and consistent dosing [43]. However, oral estrogen undergoes first-pass metabolism in the liver, which can influence its metabolism and potentially elevate clotting factors in certain individuals [43]. When prescribing oral estrogen therapy, it’s essential to take into account the patient’s medical history, liver function, and individual risk factors.

### Transdermal estrogen patches

Transdermal estrogen patches deliver estrogen directly into the bloodstream via the skin [44]. These patches offer a continuous release of estrogen, bypassing the liver’s first-pass metabolism [44]. They are particularly beneficial for individuals who encounter gastrointestinal side effects from oral formulations or have concerns regarding liver metabolism. Transdermal patches are convenient, typically needing application just once or twice a week, ensuring a consistent and steady estrogen release. However, those with certain skin conditions or sensitivities might not be ideal candidates for transdermal estrogen therapy.

### Topical estrogen creams or gels

Topical estrogen creams or gels are applied directly to the skin, facilitating estrogen absorption and systemic delivery [45]. These formulations provide dosing flexibility, as they can be adjusted based on individual needs. Topical estrogen therapy is particularly beneficial for addressing vaginal symptoms, such as dryness and discomfort [45]. It might be the preferred choice for those seeking a non-oral administration route or needing targeted relief from genitourinary symptoms. However, precautions, like washing hands after application, are essential to prevent the transfer of estrogen to others through skin contact [45].

In a meta-analysis conducted by Mohammed et al., it was observed that oral ET might be associated with a higher risk of VTE and DVT compared to transdermal ET. However, it is crucial to consider that the evidence supporting these findings is based on observational studies, which warrants low confidence. As a result, when deciding on the appropriate estrogen therapy, several factors come into play, including patient preferences, treatment objectives, and individual considerations. In this collaborative process, healthcare providers work closely with patients to determine the most suitable form of estrogen therapy that effectively addresses their needs while ensuring both convenience and safety. In essence, this patient-centric approach fosters a tailored and balanced decision-making process concerning estrogen therapy [46].

### Administration methods and dosing considerations

The administration of estrogen therapy necessitates a thoughtful consideration of the most suitable route and dosing regimen. Oral estrogen therapy is typically taken daily, with dosages determined by symptoms, medical history, and the response to treatment [47]. Transdermal estrogen patches should be applied to clean, dry skin and replaced regularly to ensure a consistent release of estrogen into the bloodstream. Topical creams or gels containing estrogen are applied to specific areas of the body, with the frequency determined by healthcare providers’ guidelines. Both the method of administration and dosing regimen are tailored to each patient’s individual needs and treatment objectives, aiming to optimize therapeutic benefits and minimize potential risks.

### Safety profile and potential risks associated with estrogen therapy

Like any medical intervention, estrogen therapy comes with a safety profile and potential risks that merit careful consideration. Generally, estrogen therapy is safe and well-tolerated, providing many postmenopausal women with significant benefits. Nevertheless, it is essential to be aware of potential risks and side effects. Common side effects of estrogen therapy include breast tenderness [48], bloating [49], and spotting or breakthrough bleeding [50, 51]. These effects are often temporary and tend to diminish over time.

When prescribing estrogen therapy, healthcare providers must consider potential risks, such as an increased likelihood of certain adverse events. Prolonged use of estrogen therapy has been linked to a higher risk of blood clots, including deep vein thrombosis [52] and pulmonary embolism [53]. Individual risk factors for clotting disorders must be assessed, and the benefits of estrogen therapy should be carefully weighed against these potential risks.

The risk of developing breast cancer from long-term estrogen therapy is another important consideration. While studies suggest a slight increase in breast cancer risk, the absolute risk remains low [54]. Factors like age, personal and family history of breast cancer, and other risk factors should be considered when determining the appropriateness of estrogen therapy.

Regular monitoring and follow-up with healthcare providers are crucial during estrogen therapy to assess patient response, manage side effects, and ensure ongoing safety and efficacy. Open and transparent communication between healthcare providers and patients is essential for shared decision making and should include comprehensive information about the potential risks and benefits of estrogen therapy.

## Impact of estrogen therapy on myocardial infarction prevention

Several observational studies and clinical trials have examined the relationship between postmenopausal hormone replacement therapy (HRT) and the risk of developing MI. One such trial, the Heart and Estrogen/Progestin Replacement Study (HERS), investigated the effectiveness and safety of HRT in preventing coronary heart disease (CHD) in women with established coronary disease [13]. The study found an initial increase in the risk of CHD events, leading to the recommendation that HRT should not be initiated for secondary prevention of CHD. However, given the favorable pattern of CHD events observed after several years of therapy, it may be reasonable for women already receiving this treatment to continue [13].

A study published in the BMJ aimed to investigate the long-term effects of synthetic 17-β-estradiol HRT on cardiovascular outcomes in recently postmenopausal women. This randomized controlled trial (RCT) involved 1006 healthy women aged 45–58, with half receiving synthetic 17-β-estradiol HRT and the other half in the control group. After approximately 11 years of intervention, women on synthetic 17-β-estradiol HRT showed a significantly reduced risk of mortality, heart failure, or MI compared to the control group. The cardiovascular benefits persisted even after 16 years, suggesting potential advantages of early synthetic 17-β-estradiol HRT in recently postmenopausal women without significant adverse events [55].

Another randomized trial, the Women’s Health Initiative (WHI), found that the use of estrogen combined with progestin was associated with higher hazard ratios for breast cancer and cardiovascular disease, including MI, in women who reported prior postmenopausal hormone use [56]. However, estrogen-only HRT was not found to be associated with an increased risk of breast cancer, as reported by the American Cancer Society [57].

The study by Tackett et al. investigated the association between HRT and in-hospital or 30-day outcomes in women presenting with acute MI (AMI). The study analyzed data from the Global Use of Strategies to Open Occluded Coronary Arteries III (GUSTO-III) trial, which included 15,059 patients, of whom 4124 were women. The researchers found that 16% of U.S.-enrolled postmenopausal women were receiving HRT, consistent with findings from the Framingham study. However, because HRT’s use was neither randomized nor stratified in the GUSTO-III trial, the study did not allow for speculation about any cardioprotective effects based on its findings [58].

A meta-analysis published in PLOS ONE reviewed 10 trials involving 38,908 postmenopausal women to assess the effects of HRT on cardiovascular outcomes. The analysis found that estrogen combined with medroxyprogesterone acetate therapy did not significantly impact coronary events, MI, cardiac death, total mortality, or revascularization compared to placebo. However, it was associated with a 27% increased risk for incident stroke. Similarly, estrogen therapy alone did not significantly affect coronary events, MI, cardiac death, total mortality, or revascularization, but it was associated with a 27% increased risk for incident stroke [59].

Another meta-analysis by Kim et al. also presented mixed findings. It evaluated the association between menopausal hormone therapy and cardiovascular disease using both RCTs and observational studies. The results indicated the increased risks of venous thromboembolism in both RCTs and observational studies. The RCTs showed an increased risk of stroke, while the observational studies suggested a decreased risk of MI. Subgroup analyses revealed varying clinical effects based on factors, such as the timing of initiation, underlying diseases, regimen types, and routes of administration [60].

Similarly, a Cochrane review of 19 trials involving 40,410 participants evaluated the effects of hormone therapy on cardiovascular disease prevention in postmenopausal women. The analysis indicated that hormone therapy did not provide protective benefits against all-cause mortality, cardiovascular death, non-fatal MI, angina, or revascularization. However, it was associated with an increased risk of stroke and venous thromboembolic events. Starting hormone therapy within 10 years of menopause was linked to lower mortality and CHD risk but an increased risk of venous thromboembolism persisted. Starting treatment more than 10 years after menopause did not significantly affect mortality or the risk of CHD but did increase the risk of both stroke and venous thromboembolism [61].

Overall, the impact of estrogen therapy on MI prevention in postmenopausal women remains a complex and evolving topic. Observational studies have suggested a potential reduction in the risk of MI with estrogen therapy, but their findings have been inconsistent and often limited by confounding variables. On the other hand, RCTs have produced conflicting results, with some studies indicating cardiovascular benefits and others reporting increased risks of adverse events, such as stroke and venous thromboembolism. Furthermore, systematic reviews and meta-analyses of multiple trials have yielded mixed findings, highlighting the need for a cautious interpretation of the available evidence. Clinicians should carefully assess the risks and benefits of estrogen therapy for each individual patient, taking into consideration factors, such as age, cardiovascular risk factors, and personal and family medical history.

## Estrogen therapy in myocardial infarction recovery

Studies have provided conflicting evidence regarding the effects of estrogen therapy on cardiac health in post-MI care. Some studies suggest potential benefits, such as improvements in cardiac remodeling and function, while others indicate limitations and possible adverse effects. For example, one study found that estrogen receptors have therapeutic potential for treating cardiac dysfunction after MI, suggesting a positive impact of estrogen therapy [62]. However, another study found that estrogen therapy worsened cardiac function and remodeling and reversed the beneficial effects of exercise training post-MI in ovariectomized female rats [63].

The use of estrogen therapy in post-MI care remains controversial due to these conflicting findings. On the one hand, a study indicated that initiating HRT following acute MI was associated with an elevated risk of subsequent cardiac events during follow-up [56]. On the other hand, estrogen deprivation postmenopause has been correlated with higher mortality and poorer prognosis after MI compared to men, underscoring the potential benefits of estrogen therapy in addressing estrogen deficiency [9].

Given the conflicting evidence, healthcare providers should exercise caution when considering the risks and benefits of estrogen therapy for individual patients in post-MI care. Factors, such as age, cardiovascular risk factors, and both personal and family medical histories, should be taken into account. Clinicians must carefully weigh these considerations in order to make well-informed decisions. Additional research is necessary to comprehensively understand the impact of estrogen therapy on cardiac health following MI.

## Current controversies and knowledge gaps

The relationship between estrogen therapy and the prevention of MI in postmenopausal women has yielded conflicting results and sparked ongoing debate. Observational studies suggest a possible reduction in MI risk associated with estrogen therapy, but results from clinical trials are mixed. For example, the HERS found an initial uptick in CHD events among women with established coronary disease using HRT [13]. As a result, HRT initiation for secondary prevention of CHD was discouraged. However, the trial also demonstrated a more favorable CHD event pattern after several years of treatment, suggesting potential benefits for women already on HRT [13].

Further insights were provided by the WHI trial, which revealed that the use of estrogen combined with progestin was linked to higher hazard ratios for both breast cancer and cardiovascular disease, including MI, among women who had previously used postmenopausal hormones [56]. Conversely, estrogen-only HRT did not show an increased risk of breast cancer, as reported by the American Cancer Society [57].

These conflicting findings have sparked debates on the effectiveness and safety of estrogen therapy in preventing MI in postmenopausal women, underscoring the need for additional research to clarify the relationship between estrogen therapy and MI risk. Key questions for future research include those related to primary prevention; most existing clinical trials have focused on the secondary prevention of CHD rather than the primary prevention of MI. The efficacy of estrogen therapy in reducing MI risk in women without established coronary disease merits further study. Moreover, variations in the formulation, dosage, and administration route of estrogen therapy across clinical trials could account for the mixed results observed. Further research is needed to ascertain the optimal formulation, dose, and route of administration that maximizes benefits while minimizing risks. The ideal timing for initiating estrogen therapy postmenopause and the appropriate treatment duration for MI prevention are also uncertain and warrant further investigation. Additionally, taking into account individual characteristics—such as age, cardiovascular risk factors, and both personal and family medical history—is essential for crafting personalized treatment plans, given that the efficacy and safety of estrogen therapy may differ among various subgroups of postmenopausal women.

To address the current controversies and knowledge gaps, future research on estrogen therapy and MI prevention in postmenopausal women should prioritize large-scale, rigorously designed prospective studies that include diverse populations. These studies should meticulously control for confounding factors and employ standardized protocols for data collection and analysis. Additionally, they should incorporate extended follow-up periods to investigate the potential long-term effects of estrogen therapy on cardiovascular health. Comparative studies that directly compare different estrogen formulations, dosages, and routes of administration are essential to determine the most effective and safest options for MI prevention. Furthermore, conducting subgroup analyses to pinpoint patient characteristics that influence the response to estrogen therapy will help identify which subgroups may derive the most benefit from treatment.

## Conclusion

The conflicting findings and controversies surrounding estrogen therapy and MI prevention have significant implications for clinical practice. Clinicians must carefully evaluate the risks and benefits of estrogen therapy for each individual patient, considering factors, such as age, cardiovascular risk factors, and personal and family medical history. Informed discussions with patients are crucial to ensure they understand the potential benefits and risks associated with estrogen therapy, thereby involving them in the decision-making process. Further research is needed to address the remaining knowledge gaps and controversies in this field.

Future studies should focus on the primary prevention of MI in postmenopausal women, investigating the effectiveness of estrogen therapy in reducing MI risk in women without established coronary disease. Research should also aim to determine the optimal formulation, dosage, and administration route of estrogen therapy to maximize benefits and minimize risks. Further investigation is needed to explore the optimal timing and duration of therapy, with the goal of establishing evidence-based guidelines. Long-term follow-up is essential for assessing the potential long-term effects of estrogen therapy on cardiovascular health. Furthermore, comparative studies directly comparing different estrogen formulations and evaluating their impact on MI risk are crucial for a comprehensive understanding.

By addressing these research gaps, clinicians can make more informed decisions and offer evidence-based recommendations concerning estrogen therapy for MI prevention. This will ultimately enhance patient care and outcomes for postmenopausal women at risk of MI.
